# Metastatic Low‐Grade Glioma Successfully Treated in a Pediatric Patient With BRAF A598_T599insI Mutation

**DOI:** 10.1002/cnr2.70309

**Published:** 2025-08-05

**Authors:** Pierluigi Calò, Safiatou Diallo, Laetitia Lebrun, Nathalie Gilis, Marco Preziosi, Pierre Leblond

**Affiliations:** ^1^ Department of Pediatric Oncology Université Libre de Bruxelles (ULB), Hôpital Universitaire de Bruxelles (HUB), Hopital Universitaire Des Enfants Reine Fabiola Brussels Belgium; ^2^ Department of Pathology Université Libre de Bruxelles (ULB), Hôpital Universitaire de Bruxelles (HUB), CUB Hôpital Erasme, Erasme University Hospital Brussels Belgium; ^3^ Department of Neurosurgery Université Libre de Bruxelles (ULB), Hôpital Universitaire de Bruxelles (HUB), CUB Hôpital Erasme, Erasme University Hospital Brussels Belgium; ^4^ Institute of Pediatric Hematology and Oncology (iHOPe) Lyon France

**Keywords:** BRAF *A598_T599insI*, BRAF mutation, dabrafenib, pediatric low‐grade glioma, targeted therapies, trametinib

## Abstract

**Background:**

Pediatric low‐grade gliomas are common brain tumors often driven by MAPK pathway alterations, including rare BRAF mutations.

**Case:**

This case report describes the first use of treatment combining dabrafenib and trametinib in a 10‐year‐old boy with pleomorphic xanthoastrocytoma harboring a BRAF A598_T599insI mutation. Surgery and chemotherapy failed, leading to metastatic progression; yet targeted therapy has achieved a sustained clinical and radiological response, lasting more than 2 years.

**Conclusion:**

This case highlights the potential of RAF/MEK inhibitors in rare BRAF‐mutated tumors and underscores the need for research to optimize treatment duration, manage side effects, and explore their role in non‐canonical mutations.

**Abbreviations**
NGS: next‐generation sequencing
PFS: progression free survival
pLGGs: pediatric low‐grade gliomas
PXA: pleomorphic xanthoastrocytoma
RANO: response assessment in neuro‐oncology criteria for gliomas

## 1 Introduction

Pediatric low‐grade gliomas (pLGGs) represent the most common brain tumors in children, accounting for approximately 30%–40% of all cases [1]. Historically, complete surgical resection has been the most reliable predictor of long‐term progression‐free survival [2]. On the contrary, residual and progressive tumors may require several lines of treatment, with chemotherapy and radiotherapy resulting in significant morbidity. Advances in understanding the molecular basis of pLGGs have paved the way for targeted therapies. Variants of the MAPK pathways gene are the most common somatic driver event of all, occurring in approximately 80% of all sporadic pLGGs [3]. Even in pLGGs without a detectable driver genomic alteration, upregulation of the RAS‐MAPK pathway is frequently detected [4]. Of all mutations and compared to wild‐type tumors, the BRAF *V600E* mutation is associated with poorer PFS and presents distinct molecular and clinical characteristics [5]. The use of target therapies such as RAF and MEK inhibitors is becoming increasingly common; in a recent phase II trial, the superiority of these drugs over standard care was proven as a successful first‐line treatment for BRAF *V600E* pLGGs [6, 7]. A significant response to targeted therapies has previously been reported in a patient with an unusual BRAF A598_T599insV mutated melanoma [8]. This report describes, for the first time, the use of dabrafenib and trametinib in a pediatric patient with a PXA harboring a rare BRAF A598_T599insI mutation. It also adds new evidence supporting the potential efficacy of targeted therapy in gliomas with non‐canonical BRAF alterations.

## 2 Results/Case Description

A 10‐year‐old boy was referred to our tertiary care center (*Hôpital Universitaire des Enfants Reine Fabiola, Bruxelles; October 2020*) following an episode of generalized absence seizure, with a subsequent brain CT scan revealing an intracranial mass. Personal and family medical history was not significant, and neuropsychological development was normal. Baseline laboratory investigations, including complete blood count, renal, liver, and metabolic function tests, were overall within normal limits for age: White blood cells 4.950/μl (4.500–13.500), Hemoglobin 13.2 g/dL (11.5–15.5), Platelets 422.000/ μl (150.000–450.000), Creatinine 0.36 mg/dL (0.39–0.73), Sodium 141 mmol/L (136–145), Potassium 3.7 mmol/L (3.4–4.7), Calcium 2.26 mmol/L (2.20–2.70) Glucose 122 mg/dL (70–100), Total bilirubin 0.17 mg/dL (< 1.2), Alanine aminotransferase 18 U/L (< 19), Aspartate aminotransferase 17 U/L (< 33), Lactate 1.1 mmol/L (< 2). The initial MRI revealed a large, heterogeneously contrast‐enhancing lesion in the left anterior and medial temporal lobe, measuring 20 mm in transverse diameter. Two additional lesions were observed: One located posterior and superior to the hippocampus, measuring 7 mm, and another at the superior‐external margin of the primary lesion, measuring 4 mm. A spinal MRI detected a solitary leptomeningeal enhancement at the cervical vertebrae two level.

The initial treatment approach involved neurosurgical intervention, including a partial left temporal lobectomy and subtotal resection of the left temporal portion. There were no postoperative complications. Histopathological analysis revealed a nodular, non‐infiltrative tumor with leptomeningeal spread and eosinophilic granular bodies. The Ki67 proliferation index was 5%. The targeted next‐generation sequencing (NGS) “clinical glioma panel” identified the non‐canonical BRAF *A598_T599insI* gene mutation (c.1795_1796insTAA, p.A598_T599insI, exon 15, 53% DNA mutated; COSMIC: COSV56201191) [9]. Conversely, the NGS panel did not identify any mutation in *ACVR1, ATRX, CDKN2A, EGFR, H3F3A, HIST1H3B, HIST1H3C, IDH1, IDH2, PDGFRA, PTEN, TERT*, or *TP53*. Due to lack of insufficient tumor material, methylation profiling was not performed. The diagnosis established was pleomorphic xanthoastrocytoma (PXA) grade 2, per the 2021 WHO classification [10].

**FIGURE 1:**
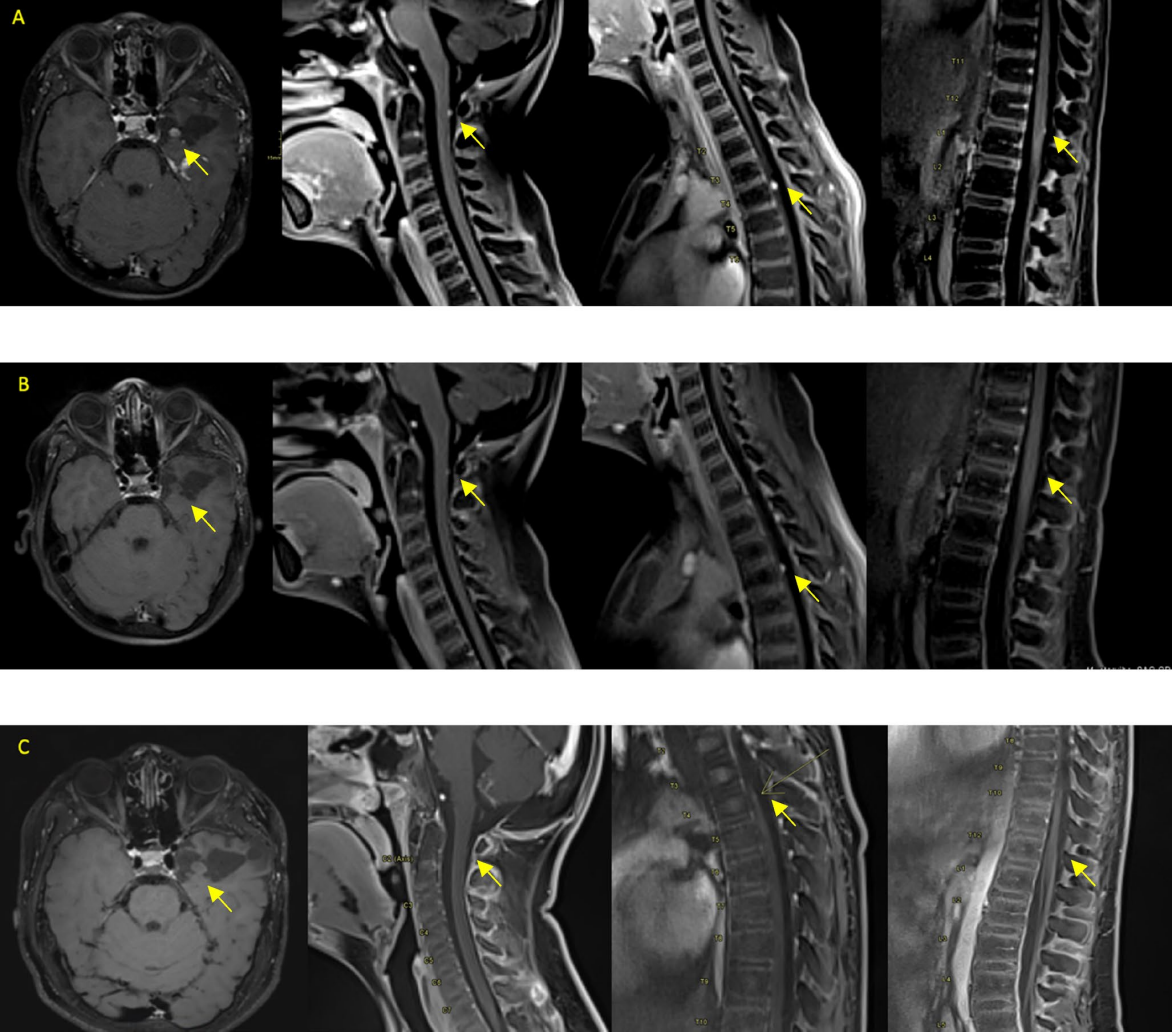
Legend, from left to right. Axial T1‐weighted brain MRI after gadolinium administration and sagittal T1‐weighted spinal MRI after gadolinium administration, performed with a 1.5 Tesla MRI scanner. (A) Baseline, prior to target therapy Brain MRI: 20 mm, 8 × 6 mm, and 1.5 mm contrast‐enhancing nodules at the margins of the resection cavity along with diffuse intracranial leptomeningeal enhancement Spinal MRI: 3.5 mm contrast‐enhancing lesion at cervical vertebrae 2, 5.7 mm at thoracic vertebrae 3, and non‐measurable lesion at thoracic vertebrae 12. (B) After 3‐months of target therapy, there was a lowering of contrast enhancement for all nodules and disappearance of the two smaller lesions. (C) After 24 months of target therapy, stability in residual size with inconsistent contrast enhancement in the bigger nodule, while the secondary lesions are almost undetectable. Nonspecific contrast enhancement of the cerebellum.

Given the favorable postoperative outcome for the patient, with a low‐grade feature and a Lansky performance score of 90%, a conservative “wait and see” approach was adopted. The institutional multidisciplinary neuro‐oncology board recommended radiological follow‐up every 3 months. Unfortunately, the patient experienced metastatic progression at the first MRI three‐month after diagnosis, with new periventricular and spinal nodular lesions. Consequently, systemic chemotherapy was initiated following the 2004 SIOP LGG Trial, combining vincristine and carboplatin [11]. The treatment was moderately tolerated, though carboplatin‐related hematologic and gastrointestinal toxicity justified a 30% dose reduction (Lansky performance score: 70%). However, post‐induction MRI 6 months after diagnosis demonstrated further disease progression with the appearance of new lesions: Two nodules measuring 8 × 6 mm and 1.5 mm at the margins of the resection cavity, along with diffuse intracranial leptomeningeal enhancement and multiple spinal lesions at cervical vertebrae 2 (3.5 mm), thoracic vertebrae 3 (5.7 mm), and thoracic vertebrae 12 (non‐measurable) levels (Figure 1A). Considering the non‐canonical activating BRAF mutation and following discussion at a multi‐institutional international pediatric neuro‐oncology tumor board, treatment was switched to a combination of oral dabrafenib and trametinib. Dabrafenib was administered at the dose of 5.25 mg/kg/day, in two equally divided doses, and trametinib at the dose of 0.025 mg/kg/day, administered once daily. Both were given in the form of tablets and respected the fasting recommendations of 2 h before and 1 h after intake. The oncological follow‐up included quarterly evaluations with a neurological assessment and a complete brain and spine MRI scan every 3 months. Monitoring of toxicity and clinical follow‐up included cardiac and ophthalmological assessments at baseline, on day 15, after 1 month, and then every 3 months for the first year, after which they were performed biannually. Laboratory investigations included a complete blood count, renal and liver function tests, and creatine phosphokinase tests monthly during treatment. Twice during follow‐up appointments, doses were adjusted according to the patient's weight.

**FIGURE 2:**
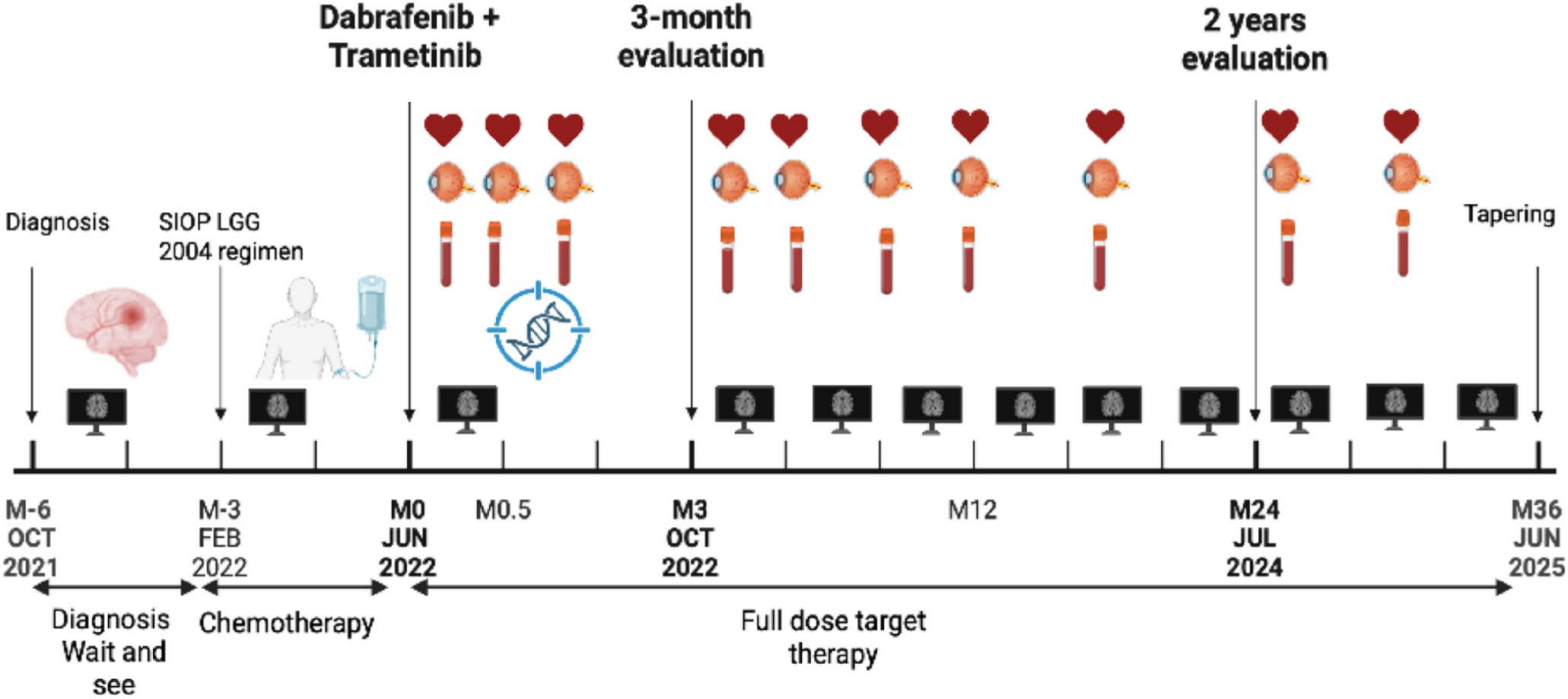
Clinical and therapeutic timeline: Schematic representation of the patient's clinical course from diagnosis through treatment with the radiological, cardiac, biological and ophtalmological follow‐up. Created with Biorender.

This approach yielded a partial response by 3 months with no new lesions, stability in the size of the larger lesion, lowering of contrast enhancement for all nodules, and the disappearance of the two smaller lesions (Figure 1B). Clinically, the patient was asymptomatic and had a Lansky score of 90%. At the two‐year follow‐up into treatment, the bigger nodule next to the surgical cavity shows stability in residual size with inconsistent contrast enhancement, while the secondary lesions are almost undetectable (Figure 1C). The most recent evaluation revealed no clinical or radiological changes. Overall, the therapy has been well tolerated (Lansky score: 90%); with no dermatological, cardiac, or ophthalmological toxicity observed. The only notable potential side effect was substantial weight gain (24.6 kg over 2 years), for which the patient has been referred to the hospital nutritionist. The case report timeline is summarized in Figure 2. The radiological assessments were consistently reviewed by a single pediatric neuroradiologist during each MRI evaluation conducted every 3 months, confirming partial response and sustained stability during the whole period of follow‐up. The standardized response assessment in neuro‐oncology criteria for gliomas (RANO) could not be applied due to the very small size of the lesions (< 10 mm) [12].

## 3 Discussion

This case highlights the potential efficacy of targeted therapies, specifically dabrafenib and trametinib, in the treatment of pLGGs with non‐canonical BRAF mutations. Although the canonical BRAF *V600E* mutation is well‐documented and widely considered as a driver mutation in pLGGs, the impact of rare mutations such as BRAF *A598_T599insI* remains poorly understood. Furthermore, there is a scarcity of data available on their clinical implications and responsiveness to targeted therapy, which is why the use of these molecules in frontline use is restricted to the *V600E* mutation [13]. The decision to use dabrafenib and trametinib was inspired by the molecular similarity between the BRAF *A598_T599insI* mutation observed in this pediatric patient and the case report of a 74‐year‐old patient with metastatic melanoma harboring a similar mutation BRAF *A598_T599insV*, for whom targeted therapy achieved a partial response lasting 5 months [8]. The BRAF mutation in the patient with a metastatic melanoma harbored a different but closely related BRAF mutation, namely p.A598_T599insV (insertion of valine), whereas our patient exhibited a p.A598_T599insI mutation (insertion of isoleucine). Although the inserted amino acids differ, both mutations occur at the same position within the kinase activation segment of BRAF, adjacent to the V600 hotspot. The potential efficacy of BRAF inhibitors on two rare BRAF mutations at the same position 599 (p.T599dup, p.A598_T599insV) has recently been validated by molecular models [14]. As shown by Bjursten et al., similar in‐frame insertions at this position alter the activation loop conformation, likely facilitating BRAF activation and supporting the rationale for targeted therapy [15]. The BRAF A598_T599insI mutation does not belong to the well‐defined Class I category of BRAF mutations, which includes RAS‐independent, monomeric, kinase‐activating variants, like *V600E*, that respond well to BRAF inhibitor monotherapy. Instead, in frame insertions within the activation segment are more consistent with Class II mutations. For this reason, a combined RAF and MEK inhibition therapy was proposed [16, 17]. Although the biological behaviors of gliomas and melanomas differ, the shared molecular pathway activation and the principle that this is as a single‐pathway disease provide a compelling rationale for this therapeutic strategy. The sustained clinical and radiological response in our patient, spanning more than 2 years, underscores the potential efficacy of RAF and MEK inhibitors in treating rare, uncommon BRAF‐mutated pLGGs. Thus far, the presence of a known BRAF alteration is an inclusion criterion for the study comparing the pan RAF inhibitor tovorafenib with the established standard of care [18].

Another noteworthy aspect of this case is the rapid metastatic disease progression following initial conservative management and systemic chemotherapy. The tumor demonstrated early metastatic potential with leptomeningeal dissemination, an atypical behavior for pleomorphic xanthoastrocytomas, which are generally more indolent. The absence of methylation profiling, currently considered a key diagnostic tool in the latest WHO classification, represents a limitation and may raise concerns about potential diagnostic uncertainty, particularly in light of the tumor's unexpectedly aggressive behavior. However, the histopathological features, clinical presentation, presence of a BRAF mutation, and the sustained therapeutic response to targeted therapy collectively support the diagnosis of a low‐grade glioma, most consistent with pleomorphic xanthoastrocytoma. This case underscores the need for a risk‐adapted treatment approach to pediatric low‐grade gliomas, guided by integrated clinical and molecular factors, as recently proposed by Ryall et al. [19].

Despite the positive therapeutic outcomes, the case also emphasizes the need for close monitoring of long‐term side effects of targeted therapies. Our patient experienced significant weight gain, one of the metabolic effects previously reported in pediatric patients receiving MEK inhibitors [13, 20]. Although manageable, these effects can have significant implications for quality of life and require a multidisciplinary approach that includes metabolic monitoring and psychosocial support, particularly to address issues such as weight‐related distress, emotional difficulties, and depression during treatment [21, 22]. Psychosocial monitoring should be an integral part of this patient's care, recognizing that targeted therapies, such as dabrafenib and trametinib, may have significant long‐term effects on the emotional and social well‐being of pediatric patients and their caregivers, despite generally being well tolerated.

This report contributes to the growing body of evidence supporting the use of targeted therapies in pLGGs with rare BRAF mutations. It also raises critical questions about the optimal duration of treatment, long‐term outcomes, and the mechanisms underlying tumor response or resistance in the context of rare mutations, as described by Pasau et al. [23]. The optimal duration of treatment for this patient has yet to be determined. However, we intend to adhere to the recent recommendations published by the Canadian group, which suggest a three‐year treatment period with subsequent dose tapering, unless significant metabolic toxicity emerges. In this case, the tapering strategy will need to be reassessed and individually adapted, especially in a patient with a “chronic” pathology [24].

In conclusion, this case illustrates the value of personalized, molecularly driven treatment in pediatric low‐grade gliomas with rare BRAF mutations. The durable response to dabrafenib and trametinib highlights the potential of targeted therapy beyond V600E mutations and supports the importance of early genetic profiling and multidisciplinary discussions. However, as this is a single‐patient case report, the findings must be interpreted with caution regarding their ability to be generalized. While the clinical and molecular features offer valuable insights, conclusions about the efficacy and long‐term outcomes of targeted therapy for rare BRAF mutations like *A598_T599insI* remain limited. Larger datasets are needed to confirm whether such responses are consistent across similarly mutated brain tumors. To address this gap, the creation of international registries for rare pediatric glioma mutations and the promotion of multi‐institutional collaborations could facilitate the collection of standardized clinical and molecular data. These efforts would enhance our understanding of BRAF variants and support evidence‐based treatment strategies in precision pediatric neuro‐oncology.

## Author Contributions

**Pierluigi Calò:** led the clinical management of the patient, conceptualized the case report, drafted, and reviewed the manuscript. **Safiatou Diallo:** contributed to patient follow‐up, data collection, and manuscript writing. **Laetitia Lebrun:** performed and interpreted the histopathological analyses, contributed to the diagnostic confirmation and manuscript revision. **Nathalie Gilis:** provided neurosurgical expertise and manuscript revision. **Marco Preziosi:** conducted and interpreted the radiological assessments throughout follow‐up and manuscript revision. **Pierre Leblond:** participated as an external expert in the international tumor board, providing key input on treatment strategy and targeted therapy selection, case management, and manuscript revision.

## Ethics Statement

All procedures performed in this study were in accordance with the ethical standards of the institutional and national research committee(s) and with the Helsinki Declaration (as revised in 2024). This case report has been approved by the Institutional Ethical Committee (HUB2025242) and written informed consent has been obtained from the patient caregiver for publication and accompanying radiological images using the Wiley patient consent form.

## Conflicts of Interest

The authors declare no conflicts of interest.

## Data Availability

The data that support the findings of this study are available from the corresponding author upon reasonable request.
